# “You should brush your teeth better”: a randomized controlled trial comparing best-possible versus as-usual toothbrushing

**DOI:** 10.1186/s12903-023-03127-3

**Published:** 2023-07-06

**Authors:** Ulrike Weik, Sadhvi Shankar-Subramanian, Thorben Sämann, Bernd Wöstmann, Jutta Margraf-Stiksrud, Renate Deinzer

**Affiliations:** 1 Institute of Medical Psychology, Justus-Liebig-University Giessen, Klinikstr. 29, Giessen, D-35392 Germany; 2 Philipps-University Marburg, Marburg, Germany; 3 Dental Clinic - Department of Prosthodontics, Justus-Liebig-University Giessen, Giessen, Germany

**Keywords:** Oral hygiene, Tooth brushing, Dental plaque, Dental health surveys, Health education, Health behavior, Periodontal disease

## Abstract

**Background:**

Most people’s tooth brushing performance is deficient, even when they are encouraged to brush to the best of their abilities. The aim of the present study was to explore the nature of this deficit by comparing best-possible vs. as-usual brushing.

**Methods:**

University students (N = 111) were randomly assigned to receive one of two instructions: “brush your teeth as usual” (AU-instruction) or “brush your teeth to the best of your abilities” (BP-instruction). Video analyses assessed brushing performance. The marginal plaque index (MPI) assessed after brushing was used as an indicator of brushing effectiveness. A questionnaire assessed subjectively perceived oral cleanliness (SPOC).

**Results:**

Participants in the BP group brushed their teeth longer (p = 0.008, d = 0.57) and used interdental devices more often (p < 0.001). No group differences emerged in the distribution of brushing time among surfaces, the percentage of brushing techniques used beyond horizontal scrubbing, or the appropriate use of interdental devices (all p > 0.16, all d < 0.30). Plaque persisted at the majority of the sections of the gingival margins, and the groups did not differ in this respect (p = 0.15; d = 0.22). SPOC values in the BP group were higher than those in the AU group (p = 0.006; d = 0.54). Both groups overestimated their actual oral cleanliness by approximately twofold.

**Conclusions:**

Compared to their usual brushing effort, study participants increased their effort when asked to brush their teeth in the best possible manner. However, that increase in effort was ineffective in terms of oral cleanliness. The results indicate that people’s concept of optimized brushing refers to quantitative aspects (e.g., longer duration, more interdental hygiene) rather than qualitative aspects (e.g., considering inner surfaces and gingival margins, appropriate use of dental floss).

**Trial registration:**

The study was registered in the appropriate national register (www.drks.de; ID: DRKS00017812; date of registration: 27/08/2019 - retrospectively registered).

**Supplementary Information:**

The online version contains supplementary material available at 10.1186/s12903-023-03127-3.

## Background

Epidemiological studies have shown the prevalence of periodontal disease in Germany and worldwide [1–4]. More than 50% of adults are affected by these chronic inflammatory disorders [4], and the global prevalence of severe periodontitis is estimated to be eleven to thirteen percent [1, 2]. One important etiological factor is persistent marginal plaque [5, 6]. Therefore, systematic oral hygiene should be performed daily to remove marginal plaque and avoid its persistent accumulation. Thorough oral hygiene is thus an important measure to prevent periodontal disease [7–9].

In line with the high prevalence of periodontal disease, however, several studies have shown that oral hygiene performance in a wide range of different age groups is inefficient in terms of oral cleanliness. Some studies also assessed brushing performance in terms of the distribution of brushing time across surfaces and sextants as well as of brushing movements [10–18]. These analyses demonstrated deficits in brushing performance, such as neglect of inner surfaces, which were present even when study participants were asked to brush to the best of their abilities.

This raises the question, what do individuals change when they clean their teeth “as good as they can” instead of cleaning the teeth “as usual”? A better understanding of this can help to uncover patients’ misconceptions toward what constitutes a very good cleaning. As a first approach to answering this question, Deinzer et al. [19] compared the brushing behavior of two cohorts of 18-year-olds examined three years apart. The first cohort had been asked to brush like usual, while the second was asked to brush to the best of their abilities. While in both cohorts the overall brushing time exceeded two minutes, the “best possible” (BP)-group spent significantly more overall time with brushing than the “as usual” (AU)-group. Most strikingly, however, the brushing pattern was very similar in both groups. Both groups neglected oral surfaces and distributed brushing time mainly across vestibular and occlusal surfaces. Furthermore, contrary to current advice, horizontal brushing was very common in both groups (40% of the brushing time of lateral surfaces).

This is a remarkable and disturbing result. This indicates that the patients’ concept of optimized brushing performance merely reflects an increase in brushing time. They do not seem to associate optimized brushing with an improvement in systematic brushing or with a change in the brushing technique in terms of brushing movements. However, prior to coming to such a conclusion, more research is needed. The former analysis [19] reflects the comparison of two cohorts studied three years apart. To come to more reliable conclusions, a randomized controlled study is needed. Such a study should also assess the effectiveness of brushing in terms of achieved oral cleanliness. It would also be of interest whether the patients do have a realistic perception of their oral cleanliness and whether the self-perceived oral cleanliness would change depending on whether one brushes as usual or to the best of one’s abilities.

Thus, the aim of the present randomized controlled study was to compare the “best possible” vs. “usual” tooth brushing with respect to (a) brushing performance, (b) subjectively perceived oral cleanliness, and (c) objectively assessed oral cleanliness after brushing. The following three research hypotheses were tested: In comparison to the “brush as usual” instruction, the “brush to the best of one’s abilities” instruction will result in the following:

- differences in brushing performance;
- better brushing outcome in terms of a higher degree of achieved oral cleanliness; and.
- a higher degree of subjectively perceived oral cleanliness.

## Materials and methods

### Study registration

This randomized controlled study has been registered at the German Clinical Trials Register (www.drks.de; ID: DRKS00017812; 2019). Registration was conducted retrospectively in August 2019 after completion of data assessment. The authors did not pay necessary attention to early registration because they were convinced that this was not a clinical trial in the true sense of the word. This was because no patients were enrolled and no health care intervention (e.g., medical or other therapeutic intervention) was applied. Discussions with colleagues on this issue raised doubts about this concept, and the trial was subsequently registered retrospectively. Prospective registration is an important safeguard against selective and biased reporting of scientific research. At the time of registration, data had not yet been analyzed or evaluated. The current research also follows a proposal for further research published by the authors earlier [19]. In this publication the need for a randomized controlled trial with the experimental and outcome variables that are the subject of the present study was discussed. Sample size calculation within the present study also refers to the results of this earlier paper. While these aspects do not excuse late registration, they should reduce concerns that this delay has created a bias in the publication of the data.

### Ethics approval

The study protocol was conducted according to the principles of the Declaration of Helsinki and was approved by the local ethics committee (date 2019/01/23; No: 254/18) of the Medical Faculty of the University of Giessen. All participants provided informed written consent and were scheduled two different appointments between April 2019 and July 2019. The study had two objectives: the comparison of brushing as usual compared to the best of one’s abilities and the analysis of the stability of the brushing behavior within an interval of two weeks. The data presented here refer to the first objective.

### Study sample

**Fig. 1:**
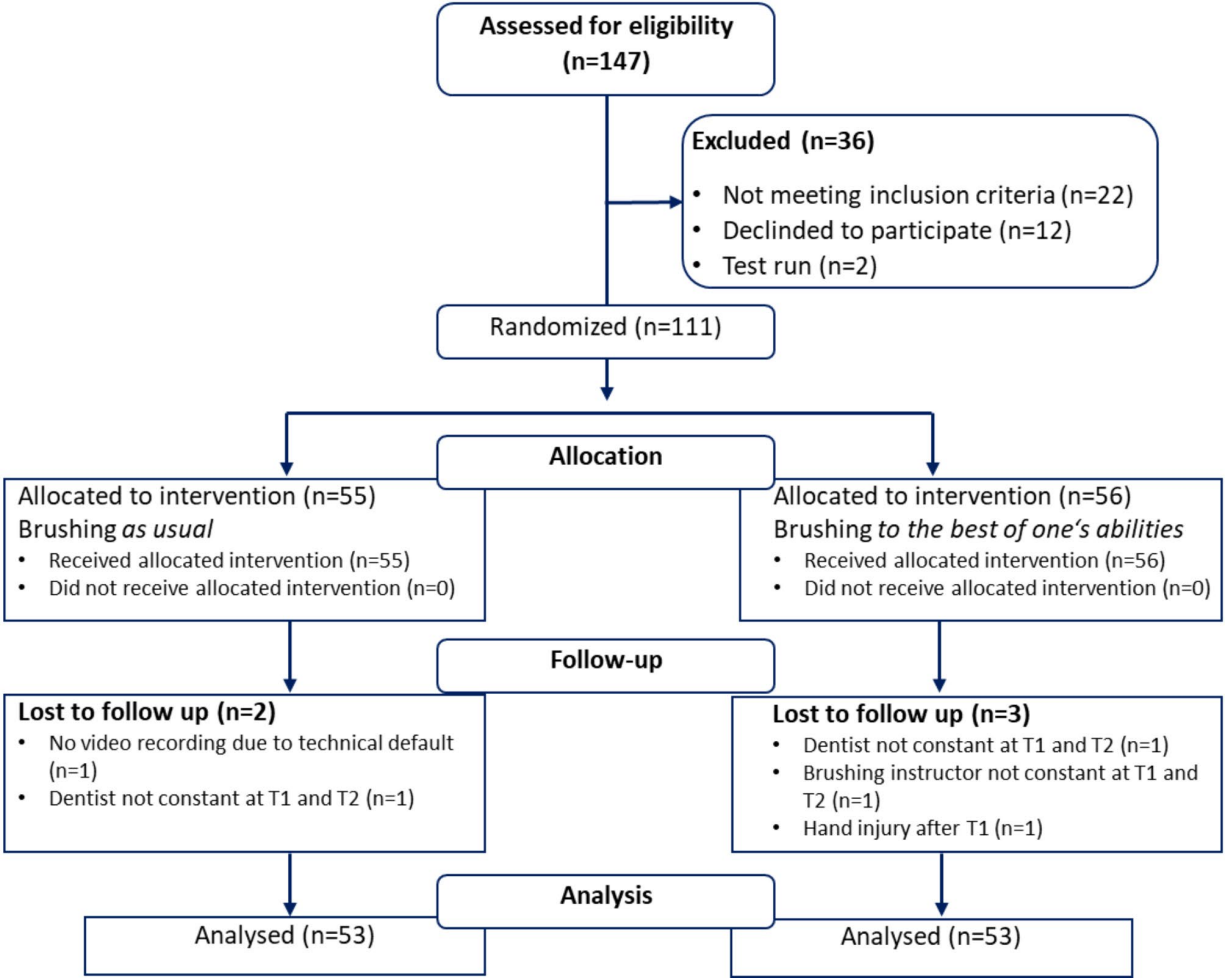
Flow diagram of participant recruitment, randomization, follow-up, and analysis

Study participants were recruited via an internal mailing list of the Justus-Liebig-University Giessen and via online announcements of a regional newspaper. Inclusion criteria were being a student-resident of Giessen aged at least 18 to a maximum of 35 years as well as the predominant use of a manual toothbrush for at least six months (at least two-thirds of all tooth brushing events). Exclusion criteria were fixed orthodontic appliances, removable prostheses/dentures, oral piercings or dental jewelry, dental prophylaxis within the previous four months, pregnancy/lactation or use of antibiotics within the previous three months, and any training in a dental or medical profession. Sample size was calculated using G*Power 3, a free available power analysis program [20], and resulted in n = 102 participants needed to detect medium effect sizes (Cohen`s d = 0.5–0.8 [21]) with α = 5% and test-power of 1–β = 0.80. With respect to behavioral parameters, such medium effect sizes were observed in a previous study [19]. To compensate for potential dropouts, we accepted up to 10% more participants over the calculated sample size. Finally, 111 participants were recruited and randomized to the study arms (see Fig. 1). All participants received a monetary compensation of 30 Euros.

### Procedure

Students interested in study participation were contacted by telephone to provide detailed information about the study, and the inclusion/exclusion criteria were checked. Eligible students were scheduled for two appointments that were two weeks apart. While study participants were asked to brush their teeth at both appointments, plaque after brushing was only assessed at the second appointment. The present study therefore focuses on the data assessed at the second appointment.

All participants were instructed to refrain from any oral hygiene behavior at least four hours before the appointments. Upon arrival at the laboratory rooms of the Institute of Medical Psychology, Justus-Liebig-University of Giessen, an assistant (A1) who was neither involved in the assessment of dental parameters nor the video recording while brushing welcomed the students and led them into the dental examination rooms. Dental plaque was assessed by one of the two dentists (TS or D2). Each dentist performed plaque assessments in 50% of the study participants. Afterward, A1 led the study participants to another room for tooth brushing where another assistant (A2) welcomed the participants. A2 accompanied them into an adjacent room equipped with a washbasin and a tablet computer with a front camera fixed at a tripod in front of the participants. This front camera served both as a mirror and as a recording tool for video recording of the participants’ tooth brushing performance. A red transparent sheet covered the surface of the tablet display to make plaque staining invisible for the participant. There were two side cameras at the walls for additional recordings used in case the tablet camera did not fully capture the brushing event. The participants were provided with a standard manual toothbrush (Elmex InterX short brush-head, medium; GABA, Loerrach, Germany) and toothpaste (Elmex; GABA, Loerrach, Germany). Additionally, dental floss (waxed and unwaxed dental floss; Elmex; GABA, Loerrach, Germany), super floss (Meridol Special-Floss; GABA, Loerrach, Germany) and interdental brushes (Elmex interdental brush sizes 2 and 4; GABA, Loerrach, Germany) were provided on a table beneath the basin. A2 informed the participants that these devices were at their free disposal. He then gave them the brushing instruction corresponding to their experimental condition (see below). Afterward, he asked them not to start brushing until they were told to do so over an intercom system. He then went to the adjacent room from which he started the video recording and repeated the respective instruction via intercom and asked them to start with tooth brushing. Participants communicated via intercom when they had finished their brushing. Immediately afterward, A2 led them back to the dental examination room where plaque was assessed again. At the end of the examination, participants were led to a neutral examination room and completed the questionnaire assessing their self-perceived oral cleanliness (SPOC) [22] as well as other questionnaires which were not within the scope of the present study. All questionnaires were presented online via a tablet computer using SoSci Survey [23] and made available to study participants at www.soscisurvey.de.

### Independent variable/experimental conditions

Participants were randomized to one out of two conditions, differing with respect to the instruction they received prior to tooth brushing. These were either “brush your teeth as thoroughly as you can so that they are completely clean” (arm 1; best of one’s abilities (BP)) or “brush your teeth as usual” “(arm 2; as usual (AU)) (instructions are translated from German; for original German instructions, see Appendix).

For randomization, A2 drew a lot with the respective instruction out of an opaque box. A2 was kept blind regarding the results of the dental examinations, as were the dentists regarding the experimental condition of the participant. To stratify with respect to participants’ sex and the examining dentist, lots were distributed to four boxes (one box for each dentist and each sex).

### Outcome variables

According to the three research hypotheses, three groups of outcomes were assessed: behavioral parameters of tooth brushing, objectively assessed dental plaque and subjectively perceived oral cleanliness.

#### a) Observed tooth brushing performance

Assessment and video analyses of the behavioral parameters were conducted according to the procedures of previous studies (e.g., [10, 24]; for a detailed description, see the Appendix) by the use of the observational software Interact 18 (Mangold International; Arnsdorf, Germany). The analyses referred to the following parameters:

Brushing parameters were as follows:

- tooth contact time;
- brushing movements (circular, horizontal, vertical, modified bass technique);
- location of the brush with respect to surfaces (outer, inner, occlusal); and
- location of the brush with respect to sextants.

Interdental hygiene parameters were as follows:

- whether a device was used and, if yes, which device was used;
- number of interdental spaces processed; and
- appropriateness of flossing technique (i.e., guiding the floss between the teeth until reaching the gum line and curving it into a C shape against one tooth to clean the proximal tooth section).

For each parameter, one examiner analyzed all videos with respect to this parameter and another who double-coded ten of the videos (see below). Altogether, seven examiners carried out the video analyses. All examiners were blinded to the experimental conditions. With respect to the clinical parameters, three examiners were completely blinded. Two of the examiners (TS, D2) were involved in the plaque assessment. To ensure their blinding as good as possible, there was a time gap of six to eight weeks between plaque assessment and video analysis. All examiners conducting the video analysis were calibrated before the beginning of the video observation. The calibration procedure was identical to previous studies (for details, see [10]): after receiving written and oral instructions from an experienced examiner (A3), all examiners were calibrated by using five videos that were not part of this study. Calibration was considered successful if an intraclass correlation (ICC) of r ≥ 0.90 was achieved with respect to all behavioral parameters of tooth brushing and interdental hygiene. All examiners achieved this criterion within the parameters they assessed. To ensure reliability during the ongoing video observations, a second examiner who was blinded to the observational results of the first examiner coded ten randomly chosen videos of the study participants. ICC analyses revealed high agreement between examiners for these double codings (total tooth contact time: ICC > 0.999; surfaces: ICC > 0.900; sextants: ICC > 0.920; brushing movements: ICC > 0.880; flossing device: ICC = 1; number of spaces: ICC = 0.982; appropriateness of flossing: ICC = 1).

The following additional parameters were calculated from the behavioral data obtained: proportional distribution of brushing time to outer, inner and occlusal surfaces (i.e., percentage of brushing time); proportional distribution of brushing time to horizontal, vertical, circular and MBT movements; and overall quality index for tooth brushing performance regarding the distribution of brushing time across sextants (QIT-S; see [19]) for outer and inner surfaces, respectively.

#### b) Objectively achieved oral cleanliness – dental plaque

An experienced dentist (D3) instructed and calibrated the examining dentists (TS, D2) prior to the study until at least 90% of the scores assessed by both examiners corresponded in five subsequent subjects (not involved in the present study) and the remaining deviated by no more than one. Dental plaque was assessed twice (before and immediately after brushing). Prior to each plaque assessment, dentists dried the teeth with an air stream and applied a plaque disclosing agent (Mira-2-Ton; Hager & Werken, Duisburg, Germany). Then, they assessed the MPI (Marginal Plaque Index, [25]) and the TQHI (Turesky modification of the plaque index of Quigley and Hein; [26]). The MPI divides the gum line into four equal sections per surface (i.e., disto-proximal, disto-cervical, mesio-cervical, mesio-proximal) and assesses the presence (score 1) or absence (score 0) of plaque within each of these sections. The overall MPI is the percentage of positive sections within all sections. The TQHI assesses the extension of plaque throughout the tooth surface. Scores range from 0 to 5: 0, no plaque; 1, flecks of stain at the gingival margin; 2, definite line of plaque at the gingival margin; 3, gingival third of surface; 4, two-thirds of surface; 5, greater than two-thirds of surface. The examining dentists were blinded to the experimental conditions. Participants were blinded to the staining of the first plaque assessment by using a red transparent sheet that covered the surface of the tablet display, which served as a mirror when they brushed their teeth. No other mirror was available until the study was finished.

#### c) Subjectively perceived oral cleanliness

To answer the question of how study participants subjectively assess their tooth brushing efficacy in terms of oral cleanliness, they completed the questionnaire for self-perceived oral cleanliness (SPOC) [22]. Subjective perception of cleanliness is assessed by a visual analog scale (VAS) presented online ranging from no cleanliness at all (score 0) to full cleanliness (score 100). First, participants naïvely estimated their overall oral cleanliness (SPOC_n_). Afterward, they receive an illustrated written explanation of the MPI assessment. They learned that, in dental judgment, a clean surface is only achieved when all plaque deposits, including those at the gum line, are removed. Then, they indicate their self-perceived oral cleanliness (SPOC_d_) according to this standard for each sextant of the outer and inner surfaces. These data inform about the self-perceived oral cleanliness according to the standards of a dentist (SPOC_d_). They also allow for a detailed analysis regarding SPOC_d_ with respect to surfaces and sextants.

### Oral health status

For clinical description of the study groups, dental status (decayed, missing and filled teeth), the Papillary Bleeding Index (PB [27] modified by Rateitschak [28]) and the periodontal screening index (PSI; [29]) were assessed prior to tooth brushing. PBI was determined at the outer and inner surfaces. Scores range from 0 to 4: 0, no bleeding on probing; 1, single bleeding point(s); 2, several bleeding points or thin line; 3, interdental triangle filled with blood; 4, profuse bleeding on probing. PSI was assessed at all teeth of each sextant by the use of a WHO probe. Scores of the PSI range from 0 to 4: 0, probing depth (PD) < 3.5 mm, no bleeding on probing, no calculus and no defective margins of restorations; 1, PD < 3.5 mm, bleeding on probing, no calculus and no defective margins; 2, PD < 3.5 mm, calculus and/or defective margins; 3, PD > 3.5 ≤ 5.5 mm with or without bleeding on probing, with or without calculus, with or without defective margins; 4, PD < 5.5 mm with or without bleeding on probing, with or without calculus, with or without defective margins. For each sextant, the highest score was recorded.

### Statistical analyses

The statistical analyses were carried out with the use of a statistical software package (IBM SPSS Statistics for Windows, Version 28; IBM, Armonk, New York, USA). Participants showing outlying values (defined as three standard deviations from the mean) in any of the behavioral parameters were excluded from final analyses to avoid distorted data. For data description, means and standard deviations and Cohen’s d as a measure of effect size were computed; in the case of skewed data, they were supplemented by quartiles and medians (shown in the Appendix). Normal distribution was tested by the Kolmogorov‒Smirnov goodness of fit test and visual inspection. For group comparisons, t tests for independent samples, exact Mann‒Whitney U tests and chi^2^ tests were calculated, as appropriate. One- and two-tailed tests were applied depending on whether the hypothesis was directed or undirected. The significance level was set at 5%. Bonferroni’s correction was applied for multiple tests within primary outcomes.

The primary outcomes of research Hypothesis a) (brushing to the best of one’s abilities will lead to a different performance) were tooth contact time and time at occlusal and outer surfaces. Tooth contact time at inner surfaces, percentages of time by which the respective surfaces were brushed, percentages of time by which specific brushing movements were applied, and the QIT-S are the secondary outcome variables.

The primary outcome of research Hypothesis b) (brushing to the best of one’s abilities will lead to a higher degree of achieved oral cleanliness) was the overall MPI. The percentage of surfaces scoring 3–5 (TQHI % 3–5 overall) was the secondary outcome variable.

The primary outcome of research Hypothesis c) (brushing to the best of one’s abilities will lead to a higher subjectively perceived oral cleanliness) was the overall SPOC_d_ score. The SPOC_d_ scores for outer and inner surfaces are secondary outcome variables. The Appendix shows all pairs of statistical hypotheses (H0/H1) for all outcome variables and the respective descriptive and inferential statistics.

## Results

One hundred and six participants finished the study (see Fig. 1). Due to outlying value(s) (M ± 3 SD) in at least one of the behavioral parameters, 15 participants were excluded from statistical analyses. Table 1 shows the demographic data and the dental and periodontal status of the participants, including plaque levels assessed before brushing. Groups did not differ with respect to any of these parameters (see Table 1). For all outcome variables assessed, detailed statistics (means, standard deviations, effect size Cohen’s d, median, interquartile range) as well as the results of respective hypothesis tests for the whole study sample including participants with outlying data are shown in the Appendix.

**Table 1﻿:** Characteristics of the sample

	Brushing *as usual (N = 45)*	Brushing to *the best of one’s ability (N = 46)*	p
	M ± SD [min, max] n/n		
*Demographic data*			
female/male	37/8	40/6	0.57
age	23.44 ± 3.0 [19, 33]	22.60 ± 2.2 [18, 28]	0.14
*Dental status (without 3rd molars)*			
Decayed teeth (0/1–2/≥3)	36/8/1	38/8/0	0.89
Missing teeth (0/1–2/≥3)	38/5/2	44/2/0	0.15
Filled teeth (0/1–5/6–9/≥10)	14/20/8/3	16/21/8/1	0.83
DMFT	3.82 ± 3.73 [0, 13]	2.63 ± 2.90 [0, 10]	0.17
*Periodontal status (including 3rd molars)*			
PBI mean	0.69 ± 0.38 [0.1, 1.6]	0.70 ± 0.40 [0.1, 2.1]	0.87
PBI % bleeding full mouth	42.68 ± 19.31 [4.5, 78.6]	41.50 ± 19.0 [7.1, 89,3]	0.77
PBI % bleeding outer surfaces	33.44 ± 22.70 [0.0, 82.1]	29.47 ± 20.36 [0.0, 85.7]	0.38
PBI % bleeding inner surfaces	51.91 ± 20.93 [9.1, 89.3]	53.54 ± 23.00 [14.3, 92.9]	0.73
Overall PSI (0/1/2/3/4)	2/6/24/13/0	0/6/32/7/1	0.20
*Plaque before tooth brushing*			
MPI overall	75.87 ± 15.29 [32.1, 99.1]	74.72 ± 14.03 [33.9, 98.2]	0.71
MPI outer surfaces	66.93 ± 20.49 [17.0, 100.0]	64.21 ± 17.39 [22.3, 97.3]	0.50
MPI inner surfaces	84.81 ± 14.24 [27.7, 100.0]	85.23 ± 14.68 [45.5, 100.0]	0.89
MPI at cervical sites	63.15 ± 20.28 [0.9, 98.2]	61.01 ± 19.02 [16.1, 96.4]	0.61
MPI at proximal sites	88.60 ± 11.60 [55.4, 100.0]	88.43 ± 11.18 [45.5, 100.0]	0.94
TQHI % 3–5 overall	46.39 ± 23.40 [1.8, 87.5]	41.69 ± 21.66 [3.6, 87.50]	0.32
TQHI % 3–5 outer surfaces	51.32 ± 28.66 [0.0, 96.4]	44.25 ± 25.24 [3.6, 92.9]	0.22
TQHI % 3–5 inner surfaces	41.45 ± 21.89 [0.0, 82.1]	39.14 ± 24.94[0.0, 85.7]	0.64

### Group differences with respect to tooth brushing performance (research Hypothesis a)

**Fig. 2﻿:**
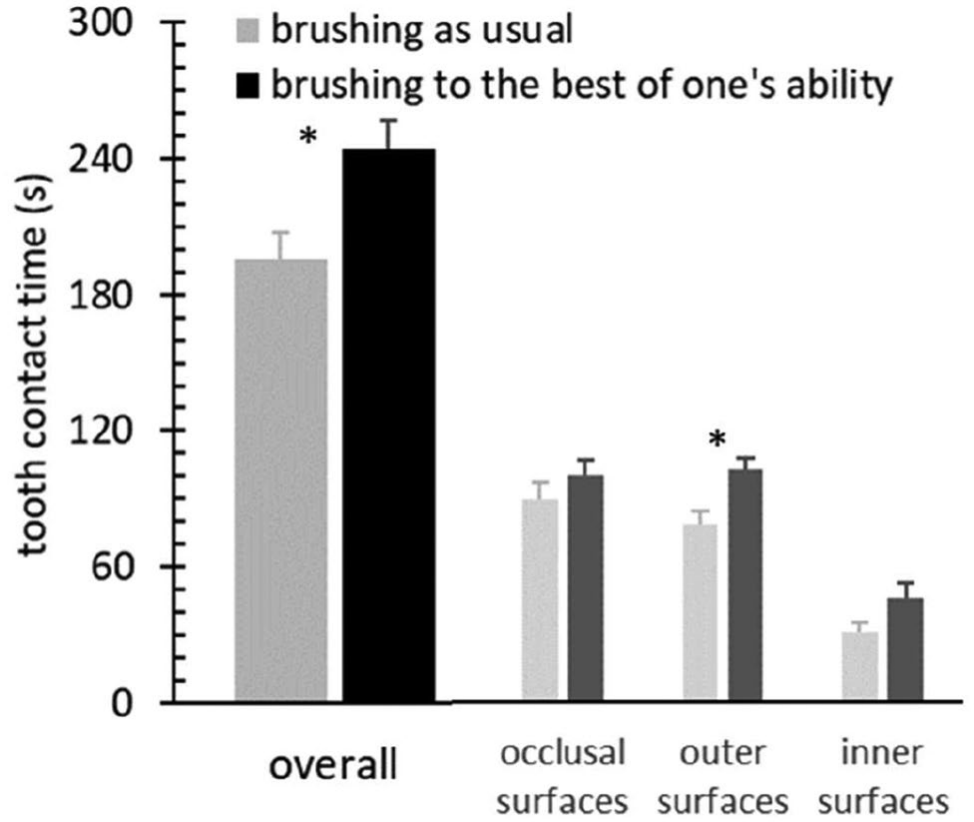
Mean and standard error of the means of observed tooth contact time (s), overall and distributed to occlusal, outer and inner surfaces, respectively. *p < 0.05

Figure 2 shows descriptive data on total tooth contact time and tooth contact time at tooth surfaces. Analysis of the primary behavioral variables, i.e., total tooth contact time and tooth contact time at the occlusal and outer surfaces, revealed significant group differences in the total tooth contact time (t_89_ = -2.700; p = 0.008; d = -0.57) and tooth contact time at the outer surfaces (t_89_ = -3.026; p = 0.003; d = -0.64). No effects were found for occlusal (t_89_ = -1.070; p = 0.288; d = -0.22) or inner surfaces (exact p = 0.159).

Data on the proportional distribution of tooth contact time to occlusal, outer and inner surfaces as well as the distribution of time spent by circular, vertical or horizontal brushing movements at lateral surfaces are shown in Table 2. No statistically significant between-group differences were found for any of these variables (see Table 2).

**Table 2:** Percentage of brushing time at teeth surfaces and brushing movements

		Brushing *as usual* (n = 45)	Brushing *to the best of one’s ability* (n = 46)	t	p	d
		Mean (SD)				
**% tooth contact time**						
Occlusal surfaces		44.60 (15.0)	40.52 (12.3)	1.421	0.16	0.30
Outer surfaces		40.63 (11.7)	42.70 (12.5)	-0.818	0.42	0.17
Inner surfaces		14.77 (10.5)	16.78 (11.7)	-0.866	0.39	0.18
**% time of different brushing movements at lateral surfaces**						
Outer surfaces^1^	Circular	64.26 (30.8)	64.85 (32.7)	-0.088	0.93	0.02
	Horizontal	33.89 (31.7)	32.41 (32.7)	0.219	0.83	0.05
Inner surfaces^2,3^	Vertical	40.23 (33.9)	30.71 (29.8)	1.361	0.18	0.30
	Horizontal	55.36 (33.0)	61.24 (33.8)	-0.803	0.43	0.18

^1^Vertical movements were rarely shown at outer surfaces and not considered for statistical analysis. ^2^ Circular movements were rarely shown at inner surfaces and not considered for statistical analysis. ^3^ Reported values refer to n = 41 vs. n = 42 within groups *as usual* vs. to the *best of one’s abilities*, respectively (n = 8 did not spend any time by brushing inner surfaces). d: effect size Cohen’s d

**Fig. 3﻿:**
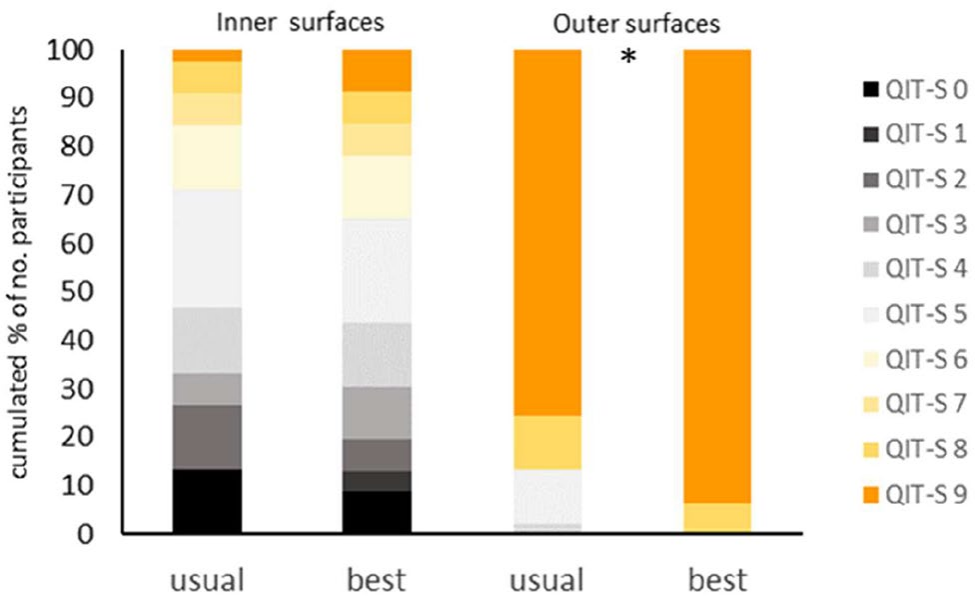
QIT-S scores at inner and outer surfaces. Scores of 0–5 indicate that 0–5 sextants were brushed for at least 1 s (brushing of less than a second is considered as neglect of the respective sextant). Score 6 indicates that every sextant was brushed for at least 1 s but less than 3.5 s, while scores 7 and 8 indicate brushing of 3.5-5 s and 5-7.5 s, respectively. A score of 9 was given when all sextants were brushed for at least 7.5 s. Usual: brushing as usual; best: brushing to the best of one’s abilities. *p < 0.05

With respect to the QIT-S (Fig. 3), statistical analyses revealed a significant effect for the overall distribution of the brushing time at outer surfaces (exact p = 0.009). No effects were found for the inner surfaces (exact p = 0.471).

### Group differences with respect to objectively achieved oral cleanliness – dental plaque after brushing (research Hypothesis b)

**Fig. 4﻿:**
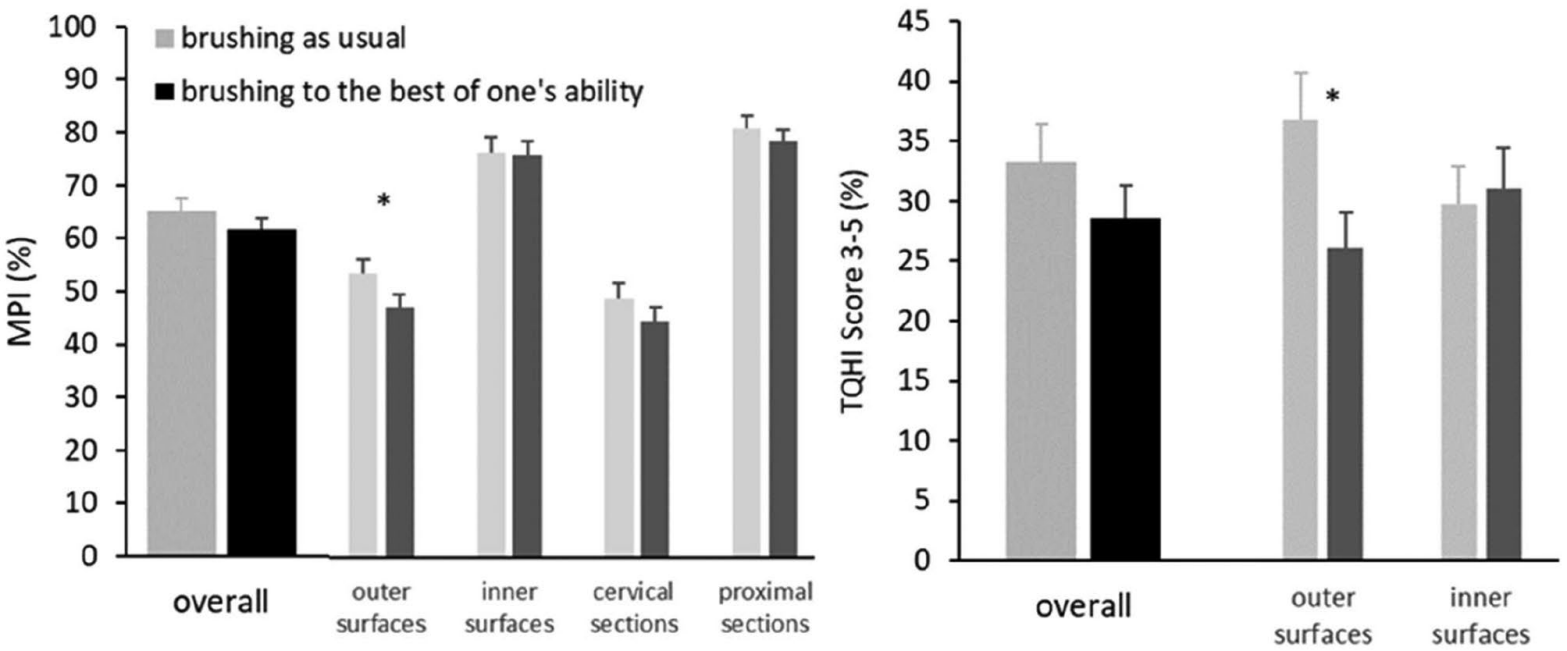
Mean and standard error of the means of plaque assessed after brushing by the Marginal Plaque Index (MPI) overall and with respect to plaque at outer, inner, cervical and proximal sections (left panel); percentage of TQHI scores 3–5 referring to rather coronal aspects of the teeth (right panel). *p < 0.05

Plaque levels after brushing are shown in Fig. 4. Groups did not differ significantly with respect to the primary (overall MPI: t_89_ = 1.045; p = 0.149; d = 0.22) or secondary (TQHI % 3–5 overall: t_89_ = 1.126; p = 0.132; d = 0.24) outcome variable. Additional analyses revealed that the largest effect sizes emerged for the outer surfaces both for the MPI (d = 0.37) and the TQHI % 3–5 (d = 0.46).

### Group differences with respect to perceived oral cleanliness (research Hypothesis c)

**Fig. 5﻿:**
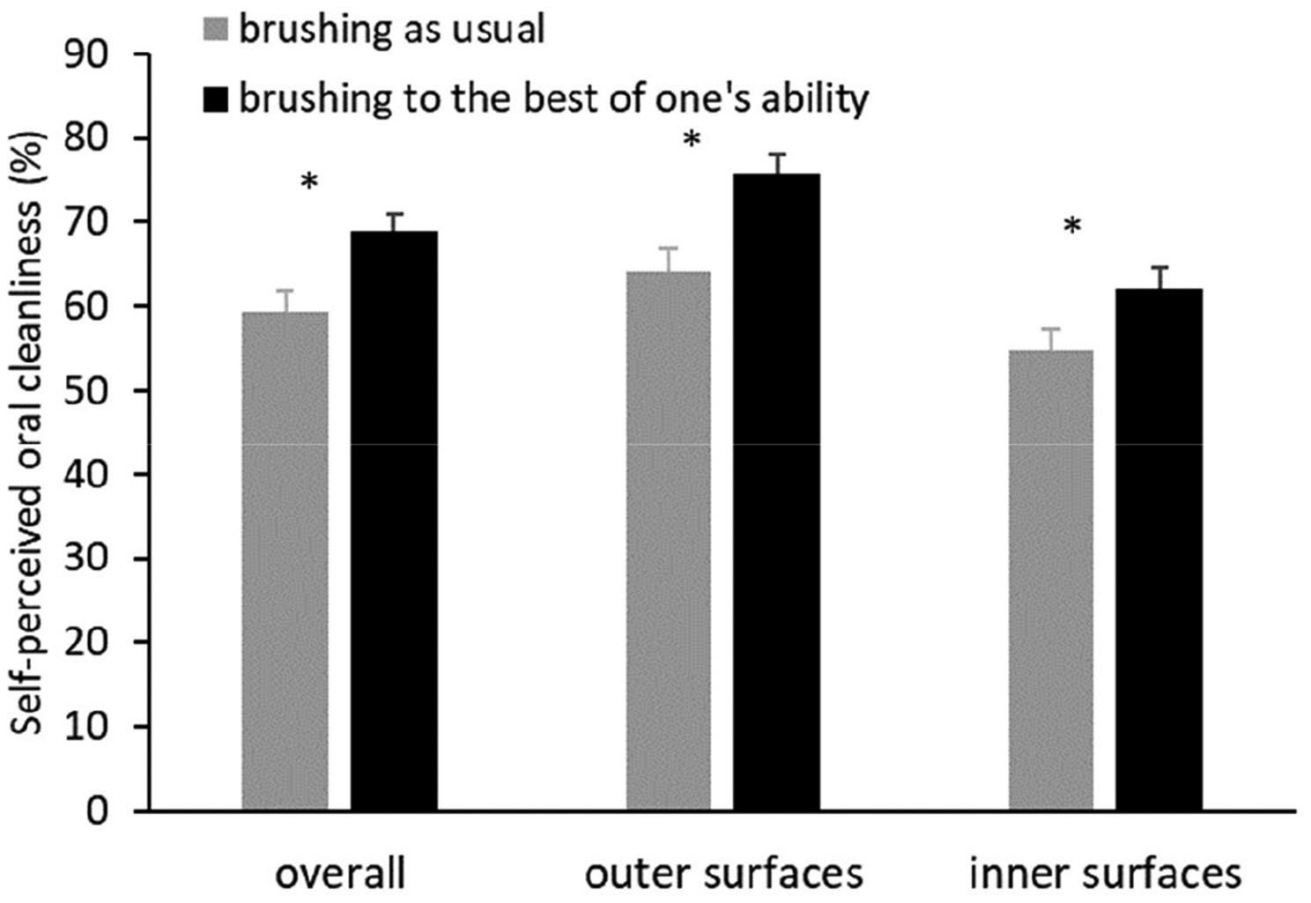
Mean and standard error of the means of the subjectively perceived oral cleanliness overall and with respect to outer and inner surfaces. *p < 0.05

Data from one study participant are missing due to temporary internet failure. Analyses of subjectively perceived oral cleanliness (Fig. 5) revealed significant group differences for the primary outcome variable, the overall SPOC_d_ (t_88_ = 2.548; p = 0.006; d = 0.54), as well as for the SPOC_d_ subscales for outer (t_88_ = 3.138; p = 0.001; d = 0.66) and inner surfaces (t_88_ = 1.824; p = 0.036; d = 0.39).

### Additional analyses

#### DMFT

Descriptive data of the DMFT show differences between the two groups, with a higher DMFT value in the AU group (Table 1). An additional analysis excluding those five participants in this group with DMFT values > 10 did not change the direction of the results. Instead, some of the effect sizes were increased (data not shown).

#### Interdental hygiene behavior

In total, n = 43 used interdental hygiene devices, with significantly more persons in the BP group than in the AU group (n = 31 vs. n = 12; exact p < 0.001). The majority of them (n = 38) applied dental floss, whereas only two of the AU group and three of the BP group applied interdental brushes. There were no group differences in the mean number of processed interdental spaces (mean ± SD: 18.42 ± 5.9 vs. 17.42 ± 6.4, respectively; p = 0.627). N = 4 in each group applied dental floss correctly.

#### Sensitivity analyses

Exclusion of outlying data led to a shortfall in the target number of evaluable subjects (n = 91 instead of n = 102). Sensitivity analyses revealed that with the current sample size, an effect size of d = 0.52 (instead of d = 0.50) would be detectable with α = 5% and a power of 1-β = 0.80 [20].

## Discussion

The instruction to brush to the best of one’s abilities led to an increased effort in the BP group. Their brushing time exceeded that of the AU group by nearly one minute (see Fig. 1). Thus, the results are in support of research Hypothesis a). Nevertheless, detailed analyses revealed that this difference had its main origin in an extended brushing of outer surfaces. The instruction did not ameliorate the neglect of inner surfaces. Instead, approximately two-thirds of both groups missed at least one sextant, and approximately 10% did not brush their inner surfaces at all. Such a neglect of inner surfaces is not a new finding [10, 11, 13–15, 18, 24, 30–32]. The present study is, however, the first to demonstrate within an RCT that the instruction to brush to the best of one’s abilities would not affect this neglect. This might be due to an important social motive of tooth brushing, i.e., removing visible plaque. The instruction to brush to the best of one’s abilities might further stimulate this social motivation. Similarly, no group differences emerged with respect to the brushing technique. The instruction to brush to the best of one’s abilities did not reduce the application of horizontal brushing movements, which are generally discouraged [33]. While strong evidence proving the superiority of specific movements is missing [9], it is remarkable that the instruction to optimize one’s brushing did not change behavior toward more elaborate brushing movements. The participants’ concept of optimized tooth brushing apparently refers mainly to the brushing quantity in terms of time but not to its quality in terms of sufficient consideration of all surfaces or the application of more elaborate brushing movements. Interestingly, the interdental cleaning behavior of the study participants also reflects this focus on quantity rather than quality. Interdental hygiene devices were used by two-thirds of the BP group compared to only one-quarter of the AU-brushers. The instruction to brush in the best possible manner increased the likelihood that participants applied interdental devices at all. Nevertheless, group membership neither made a difference in the completeness of interdental spaces processed nor in the quality of flossing. Instead, only four persons in each group performed interdental cleaning properly. Thus, the concept of thorough tooth cleaning seems to include interdental cleaning as another quantitative addition but not as an improvement in the quality of its application.

At this point, the question arises whether the increased effort shown by the BP group in terms of extended brushing time and an increased likelihood of interdental cleaning had a substantial impact on brushing success. The data are discouraging in this respect and are not in support of the research Hypothesis b). Overall plaque levels assessed immediately after brushing did not show significant group differences. Specifically, regarding the gingival margins, group differences were small. Furthermore, the more frequent use of interdental devices in the BP group did not improve their cleanliness in the proximal sections of the gum lines. Instead, plaque persisted in 80% of these sections. The toothbrush type used in this study has a crisscross design of the bristles and has been proven to be superior in its efficacy compared to other toothbrushes [34, 35]. Therefore, the high levels of remaining plaque cannot readily be attributed to an insufficient design of the toothbrush. Furthermore, the data correspond to those of earlier studies showing that even after the best possible oral hygiene, plaque would persist on most of the marginal areas [10, 13, 17, 30, 31]. They extend earlier research in that they demonstrate within an RCT that the mere advice to perform to the best of one’s abilities would not improve oral cleanliness even if people increased their effort. They also show that a mere increase in brushing time without changing other aspects of brushing behavior would not improve oral cleanliness, nor would the mere application of dental floss, since most individuals apply it improperly.

Improving one’s oral cleanliness thus requires more than an increase in brushing time and the application of tooth floss. However, people appear to have only these aspects in mind when they try to optimize their brushing behavior. In terms of dentistry, this appears to be a dysfunctional concept since it does not lead to better oral cleanliness. However, people themselves might consider it functional in that they believe that these behavioral changes would make a difference. This is exactly what the data show. The BP group rated their effectiveness even higher than the AU group. This supports research Hypothesis c). From their perspective, there appears to be no need for further changes, especially since they overestimate their oral hygiene in general. While objective plaque data indicate that less than 40% of the sections of the gingival margins were free from plaque, participants in the BP group thought it was 70%. Interestingly, both groups seem to be aware that they brush their inner surfaces less clean than their outer surfaces. Nevertheless, the BP group did not ameliorate the behavioral neglect of these surfaces in comparison to the AU group. This could indicate that during brushing, they have aspects such as time and interdental cleaning in mind rather than the oral cleanliness that they should achieve.

The current data may contribute to a better understanding of the apparent contradiction between the widespread implementation of oral hygiene as a daily routine and its low effectiveness in preventing gingivitis and periodontitis. Asking people to perform oral hygiene to the best of their abilities is a standard procedure to assess oral hygiene skills [10, 11, 13, 24, 30, 31]. For dental professionals, this request leads to almost perfect oral cleanliness [36]. They apparently perform the necessary skills. Nondental professionals apparently do not show those skills. However, they are not aware of this deficiency, as the current data show. This will most likely impede their motivation to improve their skills [37]. This lack of awareness might be due to an inappropriate concept people have regarding optimized vs. as-usual oral hygiene. Based on the results of this study, they seem to believe that optimized hygiene means increased brushing time and flossing. In fact, time and interdental hygiene are two markers of good oral hygiene that are often mentioned even in the scientific literature [8, 9]. Nevertheless, these are rather external markers of actual performance in terms of achieved oral cleanliness. They are much easier to explain and to assess than what oral hygiene is truly about: retaining the cleanliness of all surfaces and all gingival margins. This might tempt both the patients and the dental teams to focus on these external aspects when they talk about oral hygiene performance. The present data indicate that such a focus might be misleading.

The current research has certain strengths. It is a randomized controlled study, which allows for firm causal inferences. It confirms the results of an earlier less controlled quasiexperimental study regarding differences in brushing behavior with respect to the instruction to brush as usual and to brush to the best of one’s abilities [19]. It also fits with other observational data showing that people tend to neglect their inner tooth surfaces and fail to clean the areas adjacent to the gingival margins [10, 13, 17, 30, 31]. This research extends earlier findings by suggesting that increased efforts in oral hygiene would not lead to better results in terms of objective plaque levels, even though individuals might subjectively perceive their oral cleanliness to be better [22]. To prevent plaque staining at the first appointment from influencing behavior at the second appointment, plaque was not recorded at the first appointment. Despite the strengths listed above, this study also has some limitations. First, the participants were university students, and thus the data might not transfer to other populations. Nevertheless, the data are consistent with research involving other samples [10, 13, 17, 24, 30]. Second, the results focus on only one toothbrushing event. No information is available on how oral hygiene would improve if people would increase their efforts for a longer period. Third, the AU group might have increased their efforts to fulfill social norms and expectations, which might have reduced the observable differences between the two groups. The AU might have brushed longer than they would do at home. However, the data still show considerable behavioral differences in terms of time. This indicates that there was still a difference in the effort of the two groups as intended by the different instructions. Another limitation is that the overrecruitment of study participants was still insufficient. The sample size calculation resulted in a sample size of N = 102 for inferential statistics, and N = 111 were randomized. However, dropouts and outlying values resulted in only N = 91 available for inferential statistics. Nevertheless, the sensitivity analysis shows that this reduction only marginally affects the sensitivity of the statistical tests. In addition, all nonsignificant effect sizes were far below the level that could have been significant with the targeted sample size. Finally, even though plaque after oral hygiene did not differ statistically, medium effect sizes were observable with regard to more coronal parts of the crown. Nonetheless, from a clinical point of view, the differences were still small, and the overall oral cleanliness was far below what is achievable by appropriately trained people.

## Conclusion

Instruction to brush teeth to the best of one’s ability results in a greater effort compared to brushing as usual. In particular, it leads to changes in the quantitative aspects of brushing (longer duration, more interdental hygiene) but not in the qualitative aspects, such as paying attention to the inner surfaces, cleaning the gingival margins or using dental floss appropriately. However, the increase in effort goes along with an increase in self-perceived oral cleanliness, which is not verified by objective plaque assessment. Emphasizing the qualitative aspects of tooth brushing behavior and raising awareness of hygiene deficits could be a first step toward improving the effectiveness of oral hygiene.

## Electronic supplementary material

Below is the link to the electronic supplementary material.

Supplementary Material 1: Appendix

## Data Availability

The datasets used and/or analyzed during the current study are available from the corresponding author on reasonable request. However, for privacy reasons, no individual data allowing identification of participants (e.g., videos) can be provided.

## List of Abbreviations

A1: Assistant 1
A2: Assistant 2
A3: Assistant 3
AU: Experimental group (brushing as usual)
BP: Experimental group (brushing to the best of one’s abilities)
D2: Dentist 2
D3: Dentist 3
ICC: Intraclass correlation
M: Mean
MPI: Marginal Plaque Index
PBI: Papillary Bleeding Index
PD: Probing depth
PSI: Periodontal Screening Index
QIT-S: Quality index with respect to sextants
SD: Standard deviation
SPOC: Self-perceived oral cleanliness
SPOC_d_: Self-perceived oral cleanliness (assessed after participants learned that a clean surface is only achieved when all plaque deposits, including those at the gum line, are removed)
SPOC_n_: Self-perceived oral cleanliness (participants’ **n**aïve estimation)
SPSS: Statistical Package for the Social Sciences
TQHI: Turesky modification of the Plaque Index of Quigley and Hein
TS: Thorben Sämann
VAS: Visual analog scale
WHO: World Health Organization
