# Supplementary material for: “You should brush your teeth better”: a randomized controlled trial comparing best-possible versus as-usual toothbrushing
Source: BMC Oral Health. 2023 Jul 6;23:456. doi: 10.1186/s12903-023-03127-3 (PMC10327354; doi:10.1186/s12903-023-03127-3)
Supplement: Supplementary file 1 — Supplementary Material 1: Appendix [file 12903_2023_3127_MOESM1_ESM.docx]

**Appendix:**

[A: Original German brushing instruction: 2](#_Toc136544398)

[B: Description of analysis of tooth brushing performance (as described in Eidenhardt et al., 2021; BMC Oral Health, 21:359). 2](#_Toc136544399)

[C: Statistical hypotheses 3](#_Toc136544400)

[D: Descriptive and inferential statistics of brushing performance (without outlying values; N=91) 6](#_Toc136544401)

[E: Descriptive and inferential statistics of oral cleanliness before and after brushing (without outlying values; N=91) 8](#_Toc136544402)

[F: Descriptive and inferential statistics of subjectively perceived oral cleanliness (without outlying values; N=91) 10](#_Toc136544403)

[G: Descriptive and inferential statistics for the total sample (N=106) 11](#_Toc136544404)

[Sample characteristics (N=106) 11](#_Toc136544405)

[Tooth brushing performance (N=106) 12](#_Toc136544406)

[Oral cleanliness before and after tooth brushing (N=106) 14](#_Toc136544407)

[Subjectively perceived oral cleanliness (N=106) 16](#_Toc136544408)

# A: Original German brushing instruction:

Arm 1 (best of one‘s abililties): Putzen Sie Ihre Zähne so gründlich wie möglich, so, dass sie ganz sauber sind.

Arm 2 (as usual): Putzen Sie Ihre Zähne wie gewöhnlich.

# B: Description of analysis of tooth brushing performance (as described in Eidenhardt et al., 2021; BMC Oral Health, 21:359).

“Behavioural data: observed oral hygiene performance

The videos were analyzed according to the methods published by the group of Deinzer [13, 14] using the software Mangold INTERACT® 18 (Mangold International GmbH, Arnsdorf, Germany) to assess the following parameters: (1) Tooth contact time (time when the toothbrush touches the teeth, without any interruptions like spitting, rinsing etc.). (2) Tooth contact on the occlusal, inner, or outer surfaces. (3) The sextant of the tooth contact for the inner and outer surfaces. For outer surfaces the two antagonistic sextants were coded when children brushed with closed mandibles. (4) The quadrant of the tooth contact for occlusal surfaces only; (5) The brushing movements as either horizontal, vertical, circular, Modified Bass Technique or no brushing movement at all. Brushing movements were not coded at occlusal surfaces, as in general no movements other than horizontal movements were seen on these surfaces in previous studies.”

[12] Deinzer R, Ebel S, Blattermann H, Weik U, Margraf-Stiksrud J. Toothbrushing: to the best of one’s abilities is possibly not good enough. BMC Oral Health. 2018;18:167. https:// doi. org/ 10. 1186/s12903-​018-​0633-0.

[13] Deinzer R, Cordes O, Weber J, Hassebrauck L, Weik U, Kramer N, et al. Toothbrushing behavior in children: an observational study of toothbrushing performance in 12 year olds. BMC Oral Health. 2019;19:68. https:// doi. org/ 10. 1186/ s12903-​019-​0755-z.

# C: Statistical hypotheses

BP: experimental condition tooth brushing to the best of one’s abilities; AU: experimental condition tooth brushing as usual

**Primary outcome variables:**

Tooth brushing behavior:

*Tooth contact time:*

H0: µ (tooth contact time)_BP_ = µ (tooth contact time)_AU_

H1: µ (tooth contact time)_BP_ ≠ µ (tooth contact time)_AU_

The respective statistical test results in rejection of H0 in favor of H1.

*Tooth contact time at occlusal and outer surfaces:*

H0: µ (tooth contact time at occlusal surfaces)_BP_ = µ (tooth contact time at occlusal surfaces)_AU_

H1: µ (tooth contact time at occlusal surfaces)_BP_ ≠ µ (tooth contact time at occlusal surfaces)_AU_

The respective statistical test does not result in rejection of H0.

H0: µ (tooth contact time at outer surfaces)_BP_ = µ (tooth contact time at outer surfaces)_AU_

H1: µ (tooth contact time at outer surfaces)_BP_ ≠ µ (tooth contact time at outer surfaces)_AU_

The respective statistical test results in rejection of H0 in favor of H1.

Objectively achieved oral cleanliness (Marginal Plaque Index (MPI))

H0: µ (overall MPI)_BP_ ≥ µ (overall MPI)_AU_

H1: µ (overall MPI)_BP_ < µ (overall MPI)_AU_

The respective statistical test does not result in rejection of H0.

Subjectively perceived oral cleanliness (SPOC)

H0: µ (overall SPOC_d_)_BP_ ≤ µ (overall SPOC_d_)_AU_

H1: µ (overall SPOC_d_)_BP_ > µ (overall SPOC_d_)_AU_

The respective statistical test results in rejection of H0 in favor of H1.

**Secondary outcomes:**

Tooth brushing behavior:

*Tooth contact time at inner surfaces:*

H0: µ (tooth contact time at inner surfaces)_BP_ = µ (tooth contact time at inner surfaces)_AU_

H1: µ (tooth contact time at inner surfaces)_BP_ ≠ µ (tooth contact time at inner surfaces)_AU_

The respective statistical test does not result in rejection of H0.

*% tooth contact time at tooth surfaces*

Occlusal surfaces

H0: µ (% tooth contact time at occlusal surfaces)_BP_ = µ (% tooth contact time at occlusal surfaces)_AU_

H1: µ (% tooth contact time at occlusal surfaces)_BP_ ≠ µ (% tooth contact time at occlusal surfaces)_AU_

The respective statistical test does not result in rejection of H0.

Outer surfaces

H0: µ (% tooth contact time at outer surfaces)_BP_ = µ (% tooth contact time at outer surfaces)_AU_

H1: µ (% tooth contact time at outer surfaces)_BP_ ≠ µ (% tooth contact time at outer surfaces)_AU_

The respective statistical test does not result in rejection of H0.

Inner surfaces

H0: µ (% tooth contact time at inner surfaces)_BP_ = µ (% tooth contact time at inner surfaces)_AU_

H1: µ (% tooth contact time at inner surfaces)_BP_ ≠ µ (% tooth contact time at inner surfaces)_AU_

The respective statistical test does not result in rejection of H0.

*% time of different brushing movements at lateral surfaces*

Outer surfaces

H0: µ (% time circular movements at outer surfaces)_BP_ = µ (% time circular movements at outer surfaces)_AU_

H1: µ (% time circular movements at outer surfaces)_BP_ ≠ µ (% time circular movements at outer surfaces)_AU_

The respective statistical test does not result in rejection of H0.

H0: µ (% time horizontal movements at outer surfaces)_BP_ = µ (% time horizontal movements at outer surfaces)_AU_

H1: µ (% time horizontal movements at outer surfaces)_BP_ ≠ µ (% time horizontal movements at outer surfaces)_AU_

The respective statistical test does not result in rejection of H0.

Inner surfaces

H0: µ (% time vertical movements at inner surfaces)_BP_ = µ (% time vertical movements at inner surfaces)_AU_

H1: µ (% time vertical movements at inner surfaces)_BP_ ≠ µ (% time vertical movements at inner surfaces)_AU_

The respective statistical test does not result in rejection of H0.

H0: µ (% time horizontal movements at inner surfaces)_BP_ = µ (% time horizontal movements at inner surfaces)_AU_

H1: µ (% time horizontal movements at inner surfaces)_BP_ ≠ µ (% time horizontal movements at inner surfaces)_AU_

The respective statistical test does not result in rejection of H0.

*QIT-S*

H0: µ_U_ (QIT-S outer surfaces)_BP_ = µ_U_ (QIT-S outer surfaces)_AU_

H1: µ_U_ (QIT-S outer surfaces)_BP_ ≠ µ_U_ (QIT-S outer surfaces)_AU_

The respective statistical test results in rejection of H0 in favor of H1.

H0: µ_U_ (QIT-S inner surfaces)_BP_ = µ_U_ (QIT-S inner surfaces)_AU_

H1: µ_U_ (QIT-S inner surfaces)_BP_ ≠ µ_U_ (QIT-S inner surfaces)_AU_

The respective statistical test does not result in rejection of H0.

Objectively achieved oral cleanliness (TQHI % 3-5 overall)

H0: µ (TQHI % 3-5 overall)_BP_ ≥ µ (TQHI % 3-5 overall)_AU_

H1: µ (TQHI % 3-5 overall)_BP_ < µ (TQHI % 3-5 overall)_AU_

The respective statistical test does not result in rejection H0.

Subjectively perceived oral cleanliness (SPOC) for outer/inner surfaces

H0: µ (SPOC_d_ outer surfaces)_BP_ ≤ µ (SPOC_d_ outer surfaces)_AU_

H1: µ (SPOC_d_ outer surfaces)_BP_ > µ (SPOC_d_ outer surfaces)_AU_

The respective statistical test results in rejection of H0 in favor of H1.

H0: µ (SPOC_d_ inner surfaces)_BP_ ≤ µ (SPOC_d_ inner surfaces)_AU_

H1: µ (SPOC_d_ inner surfaces)_BP_ > µ (SPOC_d_ inner surfaces)_AU_

The respective statistical test results in rejection of H0 in favor of H1.

# D: Descriptive and inferential statistics of brushing performance (without outlying values; N=91)

| **Tooth contact time [seconds]** | | | | | |
| --- | --- | --- | --- | --- | --- |
|  | *Brushing*  *as usual*  *(n=45)* | *Brushing to the best of one’s abilities*  *(n=46)* |  |  |  |
|  | **Mean (SD)**  **Median (Q1, Q3)**  **Min / Max** | | **t(89)** | **p**  ***exact* p** | **d** |
| Overall | 195.43 (79.82)  198.40 (134.1, 240.7)  50.0 / 442.3 | 243.80 (90.61)  230.08 (182.8, 295.3)  103.0 / 566.8 | -2.700 | .008  .010 | -.566 |
| Occlusal surfaces | 87.66 (47.46)  81.92 (47.5, 116.8)  5.3 / 200.9 | 98.26 (47.0)  94.82 (64.2, 125.3)  24.4 / 292.4 | -1.070 | .288  .298 | -.224 |
| Outer surfaces | 77.19 (36.43)  68.16 (49.8, 105.6)  30.0 / 174.5 | 100.37 (36.6)  101.12 (71.7, 126.4)  34.3 / 173.6 | -3.026 | .003  .002 | -.635 |
| Inner surfaces | 30.57 (26.96)  26.12 (11.1, 45.0)  0.0 / 119.1 | 45.17 (42.6)  29.64 (17.1, 63.1)  0.0 / 160.2 | -1.958 | .054  .159 | -.409 |

p-value: parametrical t-test; exact p: non-parametrical Mann-Whitney-U-test; d: effect size Cohen’s d

| **Brushing time at teeth surfaces [%]** | | | | | |
| --- | --- | --- | --- | --- | --- |
|  | *Brushing*  *as usual*  *(n=45)* | *Brushing to the best of one’s abilities*  *(n=46)* |  |  |  |
|  | **Mean (SD)**  **Median (Q1, Q3)**  **Min / Max** | | **t(89)** | **P**  ***Exact* p** | **d** |
| Occlusal surfaces | 44.60 (15.02)  46.32 (36.3, 53.9)  3.0 / 71.0 | 40.52 (12.27)  41.41 (31.12, 51.79)  17.2 / 68.4 | 1.421 | .159  .169 | .298 |
| Outer surfaces | 40.63 (11.66)  40.19 (32.2, 48.0)  22.0 / 66.2 | 42.70 (12.45)  42.79 (34.2, 50.3)  18.1 / 76.4 | -.818 | .415  .353 | -.172 |
| Inner surfaces | 14.77 (10.46)  13.71 (6.4, 23.1)  0.0 / 41.9 | 16.78 (11.65)  14.75 (8.3, 26.0)  0.0 / 16.8 | -.866 | .389  .454 | -.182 |
| p-value: parametrical t-test; exact p: non-parametrical Mann-Whitney-U-test; d: effect size Cohen’s d | | | | | |

| **Time spent by different brushing movements [%]** | | | | | | |
| --- | --- | --- | --- | --- | --- | --- |
|  |  | Brushing *as usual* (n=45) | Brushing *to the best of one’s ability* (n=46) |  |  |  |
|  |  | **Mean (SD)**  **Median (Q1, Q3)**  **Min / Max** | | **t(89)** | **p**  ***exact* p** | **d** |
| **% time of different brushing**  **movements at lateral surfaces** | | |  |  |  |  |
| Outer surfaces^1^ | Circular | 64.264 (30.80)  74.28 (47.7, 90.0)  0.0 / 99.3 | 64.851 (32.74)  79.59 (37.3, 93.1)  0.0 / 100.0 | -.088 | .930  .623 | -.018 |
|  | Horizontal | 33.891 (31.70)  22.35 (9.0, 52.2)  0.0 / 100.0 | 32.414 (32.68)  19.68 (6.0, 59.3)  0.0 / 100.0 | .219 | .827  .599 | .046 |
| Inner  surfaces^2,3^ | Vertical | 40.227 (33.88)  33.10 (6.2;63.0)  0.0 / 100.0 | 30.705 (29.79)  23.85 (2.8, 46.8)  0.0 / 100.0 | 1.361 | .177  .192 | .299 |
|  | Horizontal | 55.358 (32.96)  55.72 (34.5, 85.9)  0.0 / 100.0 | 61.24 (33.8)  65.04 (36.2, 97.2)  0.0 / 100.0 | -.803 | .425  .356 | -.176 |
| ^1^Vertical movements were rarely shown at outer surfaces and not considered for statistical analysis. ^2^Reported values refer to n=41 and n=42 within conditions of brushing instruction *as usual* vs to the *best of one’s abilities*, respectively (n=8 did not spend any time by brushing inner surfaces. ^3^Circular movements were rarely shown at inner surfaces and not considered for statistical analysis.  p-value: parametrical t-test; exact p: non-parametrical Mann-Whitney-U-test; d: effect size Cohen’s d | | | | | | |

# E: Descriptive and inferential statistics of oral cleanliness before and after brushing (without outlying values; N=91)

| **Marginal Plaque Index (MPI) assessed BEFORE tooth brushing** | | | | | |
| --- | --- | --- | --- | --- | --- |
|  | *Brushing*  *as usual*  *(n=45)* | *Brushing to the best of one’s abilities*  *(n=46)* |  |  |  |
|  | **Mean (SD)**  **Median (Q1, Q3)**  **Min / Max** | | **t(89)** | **P**  ***exact* p** | **d** |
| Overall | 75.867 (15.29)  78.57 (67.0, 86.4)  32.1 / 99.1 | 74.722 (14.03)  77.68 (67.3, 85.3)  33.9/ 98.2 | .372 | .711  .485 | .078 |
| Outer surfaces | 66.935 (20.49)  73.21 (51.3, 82.7)  17.0 / 100.0 | 64.209 (17.39)  68.30 (49.1, 74.1)  22.3 / 97.3 | .685 | .495  .242 | .144 |
| Inner surfaces | 84.814 (14.24)  90.18 (78.6, 95.1)  27.7 / 100.00 | 85.233 (14.68)  87.95 (76.7, 98.4)  45.5 / 100.0 | -.138 | .890  .970 | -.029 |
| Cervical sections | 63.154 (20.28)  66.96 (51.9, 77.0)  .9 / 98.2 | 61.014 (19.02)  62.95 (48.9, 76.8)  16.1 / 96.4 | .519 | .605  .476 | .109 |
| Proximal sections | 88.599 (11.60)  92.86 (79.9, 97.3)  55.4 / 100.0 | 88.431 (11.18)  91.52 (81.9, 97.3)  45.5 / 100.0 | .071 | .944  .786 | .015 |
| p-value: parametrical t-test; exact p: non-parametrical Mann-Whitney-U-test; d: effect size Cohen’s d | | | | | |

| **Turesky’s modification of the plaque index of Quigely and Hein (TQHI) scores 3-5 (%) assessed BEFORE tooth brushing** | | | | | | | | | | |  |
| --- | --- | --- | --- | --- | --- | --- | --- | --- | --- | --- | --- |
|  | *Brushing*  *as usual*  *(n=45)* | | *Brushing to the best of one’s abilities*  *(n=46)* | |  | |  | |  | |  |
|  | **Mean (SD)**  **Median (Q1, Q3)**  **Min / Max** | | | | **t(89)** | | **p**  ***exact* p** | | **d** | |  |
| Overall | 46.390 (23.40)  43.75 (27.7, 64.3)  1.8 / 87.5 | | 41.694 (21.66)  41.07 (25.9, 57.1)  3.6 / 87.5 | | .994 | | .323  .354 | | .208 | |  |
| Outer surfaces | 51.320 (28.66)  50.0 (23.2, 77.6)  0.0 / 96.4 | | 44.249 (25.24)  42.86 (21.4, 64.3)  3.6 / 92.9 | | 1.250 | | .215  .239 | | .262 | |  |
| Inner surfaces | 41.460 (21.89)  42.86 (26.7, 57.1)  0.0 / 82.1) | | 39.139 (24.94)  37.50 (17.9, 61.6)  0.0 / 85.7 | | .471 | | .638  .536 | | .099 | |  |
|  |  | |  | |  | |  | |  | |  |
| **Marginal Plaque Index (MPI) assessed AFTER tooth brushing** | | | | | | | | | | | |
|  | | *Brushing*  *as usual*  *(n=45)* | | *Brushing to the best of one’s abilities*  *(n=46)* | |  | |  | |  | |
|  | | **Mean (SD)**  **Median (Q1, Q3)**  **Min / Max** | | | | **t(89)** | | **p**  ***exact* p** | | **d** | |
| Overall | | 65.024 (16.41)  67.41 (54.2, 79.2)  25.9 / 92.9 | | 61.615 (14.67)  62.05 (52.0, 70.6)  32.6 / 93.3 | | 1.045 | | .149  .103 | | .219 | |
| Outer surfaces | | 53.537 (18.30)  55.56 (37.5, 68.5)  10.7 / 88.4 | | 47.313 (15.74)  48.77 (33.9, 54.9)  19.6 / 89.3 | | 1.741 | | .043  .022 | | .365 | |
| Inner surfaces | | 76.510 (19.35)  80.36 (65.2, 94.2)  16.1 / 94.2 | | 75.924 (17.95)  77.23 (62.3, 89.8)  41.1 / 100.0 | | .150 | | .441  .328 | | .031 | |
| Cervical sections | | 48.950 (19.63)  52.88 (36.3, 64.3)  0.0 / 90.2 | | 44.528 (18.41)  44.64 (31.3, 55.8)  11.6 / 87.5 | | 1.109 | | .135  .094 | | .232 | |
| Proximal sections | | 81.104 (14.58)  83.04 (70.8, 95.5)  50.0 / 98.2 | | 78.702 (13.58)  80.41 (69.4, 88.4)  43.8 / 99.1 | | .209 | | .209  .192 | | .171 | |

p-value: parametrical t-test; exact p: non-parametrical Mann-Whitney-U-test; d: effect size Cohen’s d

| **Turesky’s modification of the plaque index of Quigely and Hein (TQHI) scores 3-5 (%) assessed AFTER tooth brushing** | | | | | |
| --- | --- | --- | --- | --- | --- |
|  | *Brushing*  *as usual*  *(n=45)* | *Brushing to the best of one’s abilities*  *(n=46)* |  |  |  |
|  | **Mean (SD)**  **Median (Q1, Q3)**  **Min / Max** | | **t** | **p**  ***exact* p** | **d** |
| Overall | 33.224 (21.21)  28.57 (13.5, 50.0)  0.0 / 80.4 | 28.540 (18.41)  26.79 (15.6, 43.1)  0.0 / 67.0 | 1.126 | .132  .183 | .236 |
| Outer surfaces | 36.780 (26.01)  25.00 (16.1, 60.7)  0.0 / 89.3 | 26.086 (20.12)  23.44 (10.7, 35.7)  0.0 / 75.0 | 2.197 | .015  .032 | .461 |
| Inner surfaces | 29.668 (21.83)  28.13 (10.7, 46.4)  0.0 / 75.0 | 30.994 (23.12)  25.00 (10.7, 47.3)  0.0 / 85.7 | -.281 | .390  .418 | -.059 |
| p-value: parametrical t-test; exact p: non-parametrical Mann-Whitney-U-test; d: effect size Cohen’s d | | | | | |

# F: Descriptive and inferential statistics of subjectively perceived oral cleanliness (without outlying values; N=91)

| **Subjectively perceived oral cleanliness (SPOC) [%]** | | | | | |
| --- | --- | --- | --- | --- | --- |
|  | *Brushing*  *as usual*  *(n=44)* | *Brushing to the best of one’s abilities*  *(n=46)* |  |  |  |
|  | **Mean (SD)**  **Median (Q1, Q3)**  **Min / Max** | | **t(89)** | **P**  ***exact* p** | **d** |
| Overall | 59.466 (19.99)  63.88 (46.1, 74.8)  12.2 / 93.0 | 68.976 (15.20)  70.50 (60.1, 80.0)  18.6 / 93.8 | -2.548 | .006  .007 | .537 |
| Outer surfaces | 64.239 (19.34)  69.50 (52.7, 79.3)  18.2 / 96.0 | 75.800 (15.48)  78.42 (70.5, 86.8)  17.5 / 100.0 | -3.138 | .001  .001 | .662 |
| Inner surfaces | 54.693 (21.79)  58.92 (38.3, 70.2)  3.3 / 90.0 | 62.152 (16.79)  65.00 (48.9, 74.9)  19.7 / 95.83 | -1.824 | .036  .059 | .385 |
| p-value: parametrical t-test; exact p: non-parametrical Mann-Whitney-U-test; d: effect size Cohen’s d | | | | | |

# G: Descriptive and inferential statistics for the total sample (N=106)

## Sample characteristics (N=106)

| **Characteristics of the sample (n=106)** | | | | |
| --- | --- | --- | --- | --- |
|  |  | **Brushing**  ***as usual (N=53)*** | **Brushing**  **to *the best of one’s ability (N=53)*** |  |
|  |  | **M±SD [min, max] n/n** | | **p** |
| *Demographic data* | | |  |  |
| female/male | | 45/8 | 43/9 | .80 |
| age | | 23.49 ± 3.0 [19, 33] | 22.87 ± 2.6 [18, 31] | .26 |
| *Dental status (without 3^rd^ molars)* | | |  |  |
| Decayed teeth (0/1-2/≥3) | | 40/11/2 | 44/9/0 | .74 |
| Missing teeth (0/1-2/≥3) | | 44/5/4 | 51/2/0 | .30 |
| Filled teeth (0/1-5/6-9/≥10) | | 18/24/8/3 | 20/22/9/2 | .72 |
| DMFT | | 3.74 ± 3.55 [0, 13] | 2.72 ± 3.40 [0, 16] | .13 |
| *Periodontal status (including 3^rd^ molars)* | | |  |  |
| PBI mean | | 0.66± 0.39 [0.0, 1.6] | 0.71±0.39 [0.1, 2.1] | .50 |
| PBI% bleeding full mouth | | 41.01±20.05 [1.8, 78.6] | 42.64±18.97 [7.1, 89.3] | .67 |
| PBI% bleeding outer surfaces | | 31.84±22.15 [0.0, 82.1] | 31.39±20.45 [0.0, 85.7] | .91 |
| PBI% bleeding inner surfaces | | 50.28±22.96 [3.6, 89.3] | 53.90±22.6 [12.5, 92.9] | .42 |
| Overall PSI (0/1/2/3/4) | | 2/6/31/14/0 | 0/7/36/9/1 | .32 |
| *Plaque before tooth brushing* | | |  |  |
| MPI overall | | 76.25±15.49 [33.0, 100.0] | 74.36±14.58 [33.0, 98.2] | .52 |
| MPI outer surfaces | | 68.28±20.53 [17.0, 100.0] | 65.52±17.96 [22.3, 97.3] | .46 |
| MPI inner surfaces | | 84.24±13.55 [29.5, 100.0] | 83.19±14.11 [43.8, 100.0] | .63 |
| MPI at cervical sites | | 65.10±20.35 [0.9, 100.0] | 62.08±19.55 [16.1, 96.4] | .44 |
| MPI at proximal sites | | 89.19±11.16 [55.4, 100.0] | 88.84±10.93 [45.5, 100.0] | .87 |
| TQHI% 3-5 overall | | 47.35±23.60 [1.8, 96.4] | 42.41±21.85 [3.6, 87.50] | .27 |
| TQHI% 3-5 outer surfaces | | 52.09±28.49 [0.0, 96.4] | 45.51±26.09 [3.6, 96.4] | .22 |
| TQHI% 3-5 inner surfaces | | 42.61±22.85 [0.0, 96.4] | 39.31±23.93 [0.0, 85.7] | .47 |

## Tooth brushing performance (N=106)

| **Tooth contact time [seconds]** | | | | | |
| --- | --- | --- | --- | --- | --- |
|  | *Brushing*  *as usual*  *(n=53)* | *Brushing to the best of one’s abilities*  *(n=53)* |  |  |  |
|  | **Mean (SD)**  **Median (Q1, Q3)**  **Min / Max** | | **t(104)** | **p**  ***exact* p** | **d** |
| Overall | 209.380 (94.57)  201.28 (142.0, 264.0)  50.0 / 489.3 | 273.562 (153.46)  235.36 (183.7, 311.4)  103.0 / 978.0 | -2.592 | .011  .014 | -.504 |
| Occlusal surfaces | 89.900 (50.84)  84.8 (47.5, 121.1)  5.3 / 233.8 | 106.824 (69.01)  96.84 (64.2, 132.6)  7.1 / 449.5 | -1.437 | .154  .212 | -.279 |
| Outer surfaces | 85.334 (41.02)  73.68 (50.5, 111.9)  30.0 / 188.6 | 114.511 (57.78)  104.20 (75.2, 145.3)  34.3 / 305.7 | -2.997 | .003  .004 | -.582 |
| Inner surfaces | 34.146 (32.72)  28.60 (11.1, 48.3)  0.0 / 173.4 | 52.227 (54.91)  36.20 (16.6, 69.9)  0.0 / 259.3 | -2.059 | .042  .172 | -.400 |

p-value: parametrical t-test; exact p: non-parametrical Mann-Whitney-U-test; d: effect size Cohen’s d

| **Brushing time at teeth surfaces [%]** | | | | | |
| --- | --- | --- | --- | --- | --- |
|  | *Brushing*  *as usual*  *(n=53)* | *Brushing to the best of one’s abilities*  *(n=53)* |  |  |  |
|  | **Mean (SD)**  **Median (Q1, Q3)**  **Min / Max** | | **t(104)** | **Exact p** | **d** |
| Occlusal surfaces | 42.817 (15.20)  44.91 (31.9, 52.2)  3.0 / 71.0 | 39.202 (12.80)  40.09 (29.3, 49.9)  6.5 / 68.4 | 1.324 | .188  .224 | .257 |
| Outer surfaces | 42.050 (12.33)  41.65 (33.1, 48.5)  22.0 / 79.5 | 44.095 (13.90)  44.12 (34.7, 50.8)  18.1 / 93.6 | -.801 | .425  .388 | -.156 |
| Inner surfaces | 15.133 (10.64)  15.27 (6.4, 23.1)  0.0 / 41.9 | 16.703 (11.46)  14.85 (8.0, 26.1)  0.0 / 45.7 | -.731 | .467  .533 | -.142 |
| p-value: parametrical t-test; exact p: non-parametrical Mann-Whitney-U-test; d: effect size Cohen’s d | | | | | |

| **Time spent by different brushing movements [%]** | | | | | | |
| --- | --- | --- | --- | --- | --- | --- |
|  |  | Brushing *as usual* (n=53) | Brushing *to the best of one’s ability* (n=53) |  |  |  |
|  |  | **Mean (SD)**  **Median (Q1, Q3)**  **Min / Max** | | **t(104)** | **p**  ***exact* p** | **d** |
| Outer surfaces^1^ | Circular | 62.868 (31.63)  73.98 (43.4, 90.2)  0.0, 100.0 | 63.988 (32.76)  79.15 (37.0, 93.2  0.0, 100.0 | -.179 | .858  .698 | -.035 |
|  | Horizontal | 30.214 (31.09)  19.52 (5.6, 49.5)  0.0, 100.0 | 30.435 (31.83)  17.33 (5.6, 58.4)  0.0, 100.0 | -.036 | .971  .919 | -.007 |
| Inner  surfaces^2,3^ | Vertical | 39.339 (34.81)  33.11 (5.3, 63.0)  0.0, 100.0 | 31.540 (30.78)  23.85 (2.9, 49.4)  0.0, 100.0 | 1.168 | .246  .310 | .237 |
|  | Horizontal | 52.597 (34.71)  52.42 (14.5, 85,9)  0.0, 100.0 | 59.400 (33.95)  64.34 (25.4, 96.2)  0.0, 100.0 | -.976 | .332  .307 | -.198 |
| ^1^Vertical movements were rarely shown at outer surfaces and not considered for statistical analysis. ^2^Reported values refer to n=41 and n=42 within conditions of brushing instruction *as usual* vs to the *best of one’s abilities*, respectively (n=8 did not spend any time by brushing inner surfaces. ^3^Circular movements were rarely shown at inner surfaces and not considered for statistical analysis.  p-value: parametrical t-test; exact p: non-parametrical Mann-Whitney-U-test; d: effect size Cohen’s d | | | | | | |

## Oral cleanliness before and after tooth brushing (N=106)

| **Marginal Plaque Index (MPI) assessed BEFORE tooth brushing** | | | | | |
| --- | --- | --- | --- | --- | --- |
|  | *Brushing*  *as usual*  *(n=53)* | *Brushing to the best of one’s abilities*  *(n=53)* |  |  |  |
|  | **Mean (SD)**  **Median (Q1, Q3)**  **Min / Max** | | **t(104)** | **exact p** | **d** |
| Overall | 77.134 (15.07)  78.92 (70.1, 86.6)  32.1 / 100.0 | 75.460 (14.27)  78.13 (68.8, 85.5)  33.9 / 98.2 | .587 | .558  .393 | .114 |
| Outer surfaces | 68.284 (20.53)  73.28 (52.7, 84.4)  17.0 / 100.0 | 65.520 (17.96)  69.64 (49.6, 75.4)  22.3 / 97.3 | .738 | .462  .239 | .143 |
| Inner surfaces | 85.998 (13.87)  90.18 (79.0, 97.1)  27.7 / 100.0 | 85.398 (14.47)  88.39 (76.7, 98.3)  45.5 / 100.0 | .218 | .828  .989 | .042 |
| Cervical sections | 65.100 ( 20.35)  66.96 (55.4, 78.1)  0.9 / 100.0 | 62.082 (19.55)  63.39 (50.0, 77.2)  16.1 / 96.4 | .779 | .438  .341 | .151 |
| Proximal sections | 89.186 (11.16)  92.86 (82.1, 97.8)  55.4 / 100.0 | 88.838 (10.93)  91.96 (82.5, 97.3)  45.5 / 100.0 | .162 | .872  .740 | .031 |
| p-value: parametrical t-test; exact p: non-parametrical Mann-Whitney-U-test; d: effect size Cohen’s d | | | | | |

| **Turesky’s modification of the plaque index of Quigely and Hein (TQHI) scores 3-5 (%) assessed BEFORE tooth brushing** | | | | | |
| --- | --- | --- | --- | --- | --- |
|  | *Brushing*  *as usual*  *(n=53)* | *Brushing to the best of one’s abilities*  *(n=53)* |  |  |  |
|  | **Mean (SD)**  **Median (Q1, Q3)**  **Min / Max** | | **t(104)** | **P**  ***exact* p** | **d** |
| Overall | 47.352 (23.60)  44.64 (27.7, 64.6)  1.8 / 96.4 | 42.412 (21.85)  41.07 (25.11, 57.14)  3.6 / 87.5 | 1.118 | .266  .302 | .217 |
| Outer surfaces | 52.100 (28.49)  50.00 (26.8, 77.6)  0.0 / 96.4 | 45.514 (26.09)  42.86 (21.7, 65.0)  3.6 / 96.4 | 1.239 | .218  .235 | .241 |
| Inner surfaces | 42.614 (22.85)  42.86 (26.8, 57.1)  0.0 / 96.4 | 39.310 (23.93)  39.29 (19.6, 60.7)  0.0 / 85.7 | .727 | .469  .439 | .141 |
| p-value: parametrical t-test; exact p: non-parametrical Mann-Whitney-U-test; d: effect size Cohen’s d | | | | | |

| **Marginal Plaque Index (MPI) assessed AFTER tooth brushing** | | | | | |
| --- | --- | --- | --- | --- | --- |
|  | *Brushing*  *as usual*  *(n=53)* | *Brushing to the best of one’s abilities*  *(n=53)* |  |  |  |
|  | **Mean (SD)**  **Median (Q1, Q3)**  **Min / Max** | | **t(104)** | **Exact p** | **d** |
| Overall | 65.992 (16.09)  67.41 (55.4, 79.5)  25.9 / 92.9 | 62.338 (15.63)  62.05 (51.8, 73.2)  32.6 / 95.3 | 1.185 | .119  .091 | .230 |
| Outer surfaces | 54.329 (18.44)  56.25 (37.5, 68.8)  10.7 / 88.4 | 48.664 (17.36)  48.44 (33.9, 57.1)  19.6 / 90.6 | 1.628 | .053  .026 | .316 |
| Inner surfaces | 77.654 (18.91)  80.36 (65.6, 95.1)  16.1 / 100.0 | 76.019 (18.04)  77.68 (62.1, 90.5)  41.1 / 100.0 | .456 | .325  .308 | .088 |
| Cervical sections | 50.234 (19.61)  53.00 (39.0, 66.1)  0.0 / 90.2 | 45.539 (19.58)  44.64 (31.3, 58.0)  11.6 / 91.4 | 1.234 | .110  .074 | .240 |
| Proximal sections | 81.754 (14.05)  83.04 (71.4, 95.5)  50.0 / 98.2 | 79.137 (14.02)  80.47 (69.2, 89.3)  43.8 / 99.2 | .960 | .170  .194 | .186 |

p-value: parametrical t-test; exact p: non-parametrical Mann-Whitney-U-test; d: effect size Cohen’s d

| **Turesky’s modification of the plaque index of Quigely and Hein (TQHI) scores 3-5 (%) assessed AFTER tooth brushing** | | | | | |
| --- | --- | --- | --- | --- | --- |
|  | *Brushing*  *as usual*  *(n=53)* | *Brushing to the best of one’s abilities*  *(n=53)* |  |  |  |
|  | **Mean (SD)**  **Median (Q1, Q3)**  **Min / Max** | | **t(104)** | **Exact p** |  |
| Overall | 33.368 (21.76)  28.57 (13.5, 50.0)  0.0 / 89.3 | 29.294 (18.93)  26.79 (15.2, 44.2)  0.0 / 67.9 | 1.028 | .153  .219 | .200 |
| Outer surfaces | 36.345 (25.50)  25.00 (17.9, 60.4)  0.0 / 89.3 | 27.938 (21.55)  25.00 (10.7, 37.5)  0.0 / 82.1 | 1.833 | .035  .055 | .356 |
| Inner surfaces | 30.390 (23.63)  25.00 (10.7, 46.4)  0.0 / 100.0 | 30.649 (22.63)  25.00 (10.7 / 46.4)  0.0 / 85.7 | -.057 | .477  .446 | -.011 |
| p-value: parametrical t-test; exact p: non-parametrical Mann-Whitney-U-test; d: effect size Cohen’s d | | | | | |

## Subjectively perceived oral cleanliness (N=106)

| **Subjectively perceived oral cleanliness (SPOC) [%]** | | | | | |
| --- | --- | --- | --- | --- | --- |
|  | *Brushing*  *as usual*  *(n=52)* | *Brushing to the best of one’s abilities*  *(n=53)* |  |  |  |
|  | **Mean (SD)**  **Median (Q1, Q3)**  **Min / Max** | | **T(103)** | **Exact p** |  |
| Overall | 60.928 (20.02)  65.67 (47.2, 76.1)  12.2 / 93.0 | 67.448 (16.06)  70.25 (57.4, 79.3)  16.7 / 93.8 | -1.843 | .034  .050 | -.360 |
| Outer surfaces | 65.212 (19.32)  70.67 (56.0, 81.1)  18.2 / 96.0 | 73.843 (16.83)  74.33 (68.0, 85.5)  17.5 / 100.0 | -2.442 | .008  .010 | -.477 |
| Inner surfaces | 56.644 (21.85)  62.17 (41.2, 74.9)  3.3 / 90.0 | 61.054 (17.16)  64.83 (48.6, 73.5)  14.7 / 95.8 | -1.151 | .126  .225 | -.225 |
| p-value: parametrical t-test; exact p: non-parametrical Mann-Whitney-U-test; d: effect size Cohen’s d | | | | | |
